# Marine Algal Toxins and Public Health: Insights from Shellfish and Fish, the Main Biological Vectors

**DOI:** 10.3390/md22110510

**Published:** 2024-11-10

**Authors:** Kuan-Kuan Yuan, Hong-Ye Li, Wei-Dong Yang

**Affiliations:** Key Laboratory of Aquatic Eutrophication and Control of Harmful Algal Blooms of Guangdong Higher Education Institute, College of Life Science and Technology, Jinan University, Guangzhou 510632, China; yk9723@stu2019.jnu.edu.cn (K.-K.Y.); thyli@jnu.edu.cn (H.-Y.L.)

**Keywords:** harmful algal blooms, diarrhetic shellfish poisoning, paralytic shellfish poisoning, neurotoxic shellfish poisoning, domoic acid, ciguatera, public health

## Abstract

Exposure to toxigenic harmful algal blooms (HABs) can result in widely recognized acute poisoning in humans. The five most commonly recognized HAB-related illnesses are diarrhetic shellfish poisoning (DSP), paralytic shellfish poisoning (PSP), amnesic shellfish poisoning (ASP), neurotoxic shellfish poisoning (NSP), and ciguatera poisoning (CP). Despite being caused by exposure to various toxins or toxin analogs, these clinical syndromes share numerous similarities. Humans are exposed to these toxins mainly through the consumption of fish and shellfish, which serve as the main biological vectors. However, the risk of human diseases linked to toxigenic HABs is on the rise, corresponding to a dramatic increase in the occurrence, frequency, and intensity of toxigenic HABs in coastal regions worldwide. Although a growing body of studies have focused on the toxicological assessment of HAB-related species and their toxins on aquatic organisms, the organization of this information is lacking. Consequently, a comprehensive review of the adverse effects of HAB-associated species and their toxins on those organisms could deepen our understanding of the mechanisms behind their toxic effects, which is crucial to minimizing the risks of toxigenic HABs to human and public health. To this end, this paper summarizes the effects of the five most common HAB toxins on fish, shellfish, and humans and discusses the possible mechanisms.

## 1. Introduction

Harmful algal blooms (HABs) generally refer to the proliferation of toxic and non-toxic algae that pose a threat to humans, aquatic organisms, and/or aquatic environments [1,2]. At high cell densities, non-toxic algae may deplete dissolved oxygen, thereby causing harm to the environment and aquatic organisms [3,4]. In this article, the term “HABs” specifically relates toxigenic HABs only to those algal species that produce toxins and pose a threat to human health. The serious impact of HABs on the safety of aquatic organisms and public health is increasingly appreciated with the dramatic rise in the number, frequency, and intensity of HABs in coastal areas around the world [5]. To date, the public Harmful Algae Events Database (HAEDAT, http://haedat.iode.org/, accessed on 5 November 2024) has more than 10,000 global data records.

In recent years, many toxic algal species and phycotoxins have been studied, and more geographic areas are affected than ever before. The proliferation and geographic dispersal of these HAB-related algae are generally attributed to natural environmental factors, anthropogenic activities, and climatic change [6,7,8]. Outbreaks of HABs may result in the death of many marine organisms, including fish and shellfish, and cause illness and even death in humans through the food chain [9,10,11]. As the demand for seafood rises, coastal urbanization accelerates, and more people engage in marine recreational activities, the risk of human illness associated with HABs is increasing [12,13,14]. As a result, a growing number of studies have focused on the toxicological assessment of HAB-related species and their toxins to aquatic organisms. However, due to the HAB toxins diversity and the complexity of toxic mechanisms, the toxicity to different aquatic organisms varies widely [10,15,16,17,18], which makes it challenging to monitor the adverse effects of HABs on these organisms and complicates the assessment of the potential threat of HABs to human health.

The consumption of seafood has become one of the main causes of food-borne diseases with a known etiology, including poisoning, allergies, and infections [19]. Additionally, HAB-related toxins are a significant source of seafood contamination globally, posing a serious threat to marine organisms, consumer health, and ecosystems [19,20]. The primary human health concerns associated with HABs stem from the consumption of fish and shellfish contaminated with these toxins [21,22]. A comprehensive review of the adverse effects of HABs on aquatic organisms can, therefore, advance our understanding of the mechanisms underlying their toxic effects and inform the development of effective public health measures to mitigate the threat to human health.

Based on the nature of the toxins associated with HABs and the symptoms of human intoxication, these toxins can be categorized into the following groups: diarrhetic shellfish poisoning (DSP) toxins, paralytic shellfish poisoning (PSP) toxins, amnesic shellfish poisoning (ASP) toxins, neurotoxic shellfish poisoning (NSP) toxins, and ciguatera poisoning (CP) toxins. This article outlines the effects of the five most common HAB toxins on aquatic organisms and humans. The aim is to summarize the current research status and suggest directions for future studies that seek to mitigate the impacts of HABs on human health.

## 2. Phycotoxins

### 2.1. DSP Toxins

#### 2.1.1. DSP Toxins: Physicochemical Properties and Structure

**Figure 1:**
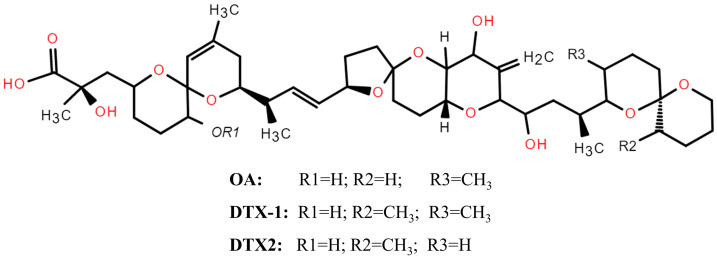
General chemical structure of DSP toxins.

DSP toxins are mainly produced by two marine dinoflagellate genera, *Dinophysis* and *Prorocentrum* [23]. Initially, the DSP toxin group included okadaic acid (OA), dinophysistoxins (DTXs), and their derivatives, as well as yessotoxins (YTXs) and pectenotoxin (PTXs) [24]. However, since 2002, YTXs and PTXs have been excluded from the DSP toxin classification, as they have been demonstrated to cause liver necrosis and myocardial damage but not diarrhea [25]. Currently, only OA, DTX-1, DTX-2, and its C-7 acyl derivative (DTX-3), which are found in shellfish [26,27,28], have been confirmed to cause diarrheal effects [29,30]. The LD_50_ values for the main DSP toxins in mice (intraperitoneally) are 185.6 µg kg^−1^ for OA, 150.4 µg kg^−1^ for DTX-1, and 352 µg kg^−1^ for DTX-2 [31]. These toxins exhibit lipophilic properties, accumulating in adipose tissues and being metabolized and eliminated with difficulty [32]. The principal action mechanism of DSP toxins is the inhibition of serine/threonine protein phosphatases 1 and 2A (PP1 and PP2A), resulting in the deregulation of numerous intracellular processes [33]. Structurally, OA and DTXs share the same carbon backbone, the differences among various analogs are only in the number or position of methyl groups (see Figure 1) [34]. Moreover, as polyether compounds, DSP toxins are fat-soluble and thermally stable [35,36]. Given its lipophilicity, DSP toxins easily accumulate in the tissues of filter-feeding marine organisms that ingest toxic algae, which may result in direct or indirect transmission to humans through the food chain [37]. Thermal stability allows them to remain active even after exposure to cooking temperatures [38]. Since the first DSP incident was reported in 1961, DSP incidents have been continuously occurring worldwide [39]. 

#### 2.1.2. Main Biological Vectors of DSP Toxins

##### Filter-Feeding Bivalves

DSP is characterized by the onset of acute gastrointestinal symptoms such as nausea, abdominal pain, and diarrhea after consumption of seafood contaminated with OA and DTXs [40,41]. Filter-feeding bivalves, including mussels, scallops, and oysters, are recognized as significant vectors of DSP toxins. DSP toxins and related HAB species are known to induce a number of physiological changes in these bivalves, including valve closure response, pseudo-feces production, and clearance rate reduction [42,43,44,45]. In addition, DSP toxins have been shown to cause histopathological changes as well as immunotoxic, cytotoxic, and genotoxic effects [46,47,48]. Despite all these biological effects, no mortality has been documented in bivalves naturally exposed to HAB species associated with DSP toxins. Many studies have shown that bivalves are tolerant to DSP toxins compared to human susceptibility, as evidenced by the fact that the toxicity of DSP toxins to bivalves decreases with exposure time [46,48,49,50].

Studies have demonstrated that after exposure to the DSP toxin-producing dinoflagellate *D. acuminata* or *D. acuta*, cytochrome 450 (CYP450) enzyme activity in the digestive gland tissues of *Mytilus galloprovincialis* significantly increased. This increase was negatively correlated with the accumulated OA content in the tissue, suggesting the potential involvement of the CYP450 enzyme system in the metabolism of DSP toxins in mussels [51]. In addition, after exposure to *P. lima*, differential expression of *CYP3A4*, *CYP2D14*, *CYP3L3*, and *CYP2C8* was observed in the digestive gland and gill tissues of *Perna viridis*. Inhibition of CYP3A4 activity significantly altered the metabolic processing of DSP toxins in the mussel, indicating a pivotal role for CYP3A4 in the metabolism of DSP toxins [52]. After exposure of *Crassostrea ariakensis* to *P. lima*, glutathione S-transferase-ω (*GST-ω*) in gill tissues and *GST-α* in digestive gland tissues were significantly up-regulated, suggesting that different isoforms of GST in different tissues are involved in the metabolic process of DSP toxins [53]. Exposure to *P. lima* can significantly induce the expression of ATP-binding cassette (ABC) transporters in mussels, with different expression levels among different isoforms and tissues [54,55]. The P-glycoprotein (P-gp) specific inhibitor significantly decreased the activity of P-gp in the gill tissues of *P. viridis* but did not affect the accumulation of DSP toxins in gill tissue. In contrast, a broad-spectrum inhibitor of ABC transporters significantly increased the accumulation of DSP toxins in gill tissues [54,55,56]. These findings indicate that different isoforms of ABC transporters, including P-gp and multidrug resistance-associated protein (MRP), may collaborate to form a complex defense network during the metabolism of DSP toxins in bivalve species [55,56,57]. 

In bivalves, the digestive gland is the main organ for the accumulation of DSP toxins, where DSP toxins undergo a transformation during the digestive process [58]. Studies have shown that free DSP toxins (OA, DTX1, and DTX2) can be esterified with fatty acids of varying carbon chain lengths to form acyl derivatives such as DTX3 [26,27,59], which has lower affinity to target proteins, resulting in reduced toxicity [60]. This may also contribute to the higher tolerance of bivalves to DSP toxins. In addition, exposure to *P. lima* can activate the Nrf2 signaling pathway in the gill and digestive gland tissues of bivalves, thereby mitigating the impact of DSP toxins [49,61]. The addition of DHA can significantly enhance the expression of genes and enzyme activity related to the Nrf2 signaling pathway and reduce the damage caused by DSP toxins to the digestive gland of *P. viridis*. This further confirms the crucial role of the Nrf2 signaling pathway in the response and detoxification process of bivalves to DSP toxins [62]. However, the molecular mechanisms regulating the Nrf2 signaling pathway during the detoxification of DSP toxins in bivalves remain unclear. Taken together, the current findings suggest that bivalves may have developed cytoprotective mechanisms to mitigate the deleterious effects of DSP toxins, including the esterification of DSP toxins, the up-regulation of the three-phase metabolic detoxification, and the antioxidant system.

##### Fish

In addition to bivalves being the main vectors of DSP toxins, fish, as an important marine biological group, have been shown to be exposed to DSP toxins through their diet [16]. However, unlike bivalve species, fish appear to be susceptible to DSP toxins. Studies have shown that exposure of larvae of *Minidia beryllina* and *Cyprinodon variegatus* to DSP toxin-producing dinoflagellates results in adverse effects on growth, feeding rates, and swimming activity [63,64]. The juveniles and adult fish exposed to DSP toxins experience oxidative stress, multiorgan histopathology, behavioral changes, and even death [65,66]. In terms of the toxicological effects of OA and DTX1 in medaka and zebrafish models, medaka fish embryo exposure to concentrations of 0.75 µg mL^−1^ of OA and toxic extract containing DSP toxins, with a concentration of 0.22 µg OA eq mL^−1^, resulted in 100% mortality [67]. Conversely, juvenile zebrafish exposed to sublethal concentrations of OA or DTX-1 exhibited developmental delays, significant pericardial edema, monocular malformations, reduced anterior–posterior axes, and oxidative stress [68]. In addition, Neves et al. (2020) assessed the sublethal toxicity of DSP toxins in fish through a food chain transfer experiment and revealed that brine shrimp were effectively used as a link for the transfer of DSP toxins to higher trophic levels, leading to the accumulation of DSP toxins in wild ringneck blenny (*Parablennius pilicornis*: Blenniidae) [66]. Although current data suggest that exposure to DSP toxins induces a variety of adverse effects in fish, including behavioral and morphological changes and increased mortality, information regarding the lethal and sublethal impacts, as well as chronic effects of HAB species associated with DSP toxins on fish in the aquatic environment is still very scarce [63,66,69], which should be of concern.

#### 2.1.3. The Mechanism and Possible Pathway of Toxicity to Humans

DSP toxins can accumulate in marine organisms and subsequently be transferred through the food chain to higher trophic levels, including humans [70]. The clinical symptoms of human poisoning by DSP toxins are mainly incapacitating diarrhea, along with gastrointestinal discomfort, nausea, vomiting, abdominal pain, and sometimes headache, chills, and fever [70]. Symptoms of DSP are very similar to those of gastrointestinal illness and can occur a few hours after consumption of contaminated seafood. Acute symptoms usually disappear within 2 to 3 days, which may lead to DSP incidents often being overlooked, so the actual DSP incidence may be much higher than that reported in the data [71]. The chronic toxicity of DSP toxins is of greater concern due to their carcinogenicity. Several studies have associated chronic exposure to DSP toxins with an increased risk of cancer, including gastrointestinal, pancreatic, and liver cancers [72,73,74]. Notably, most studies attribute the toxic mechanism of DSP toxins to the inhibition of serine/threonine protein phosphatases, specifically PP1 and PP2A; however, this inhibition does not fully account for all toxic effects [75]. Recently, studies have shown that OA exposure can cause the dysregulation of the intestinal microenvironment in rats by altering gut microbiota composition and disrupting the epithelial barrier. This suggests that OA-degrading bacteria and opportunistic pathogens play a non-negligible role in OA toxicity [76,77], resulting in the complex toxicity profile of DSP toxins.

### 2.2. PSP Toxins

#### 2.2.1. PSP Toxins: Physicochemical Properties and Structure

**Figure 2:**
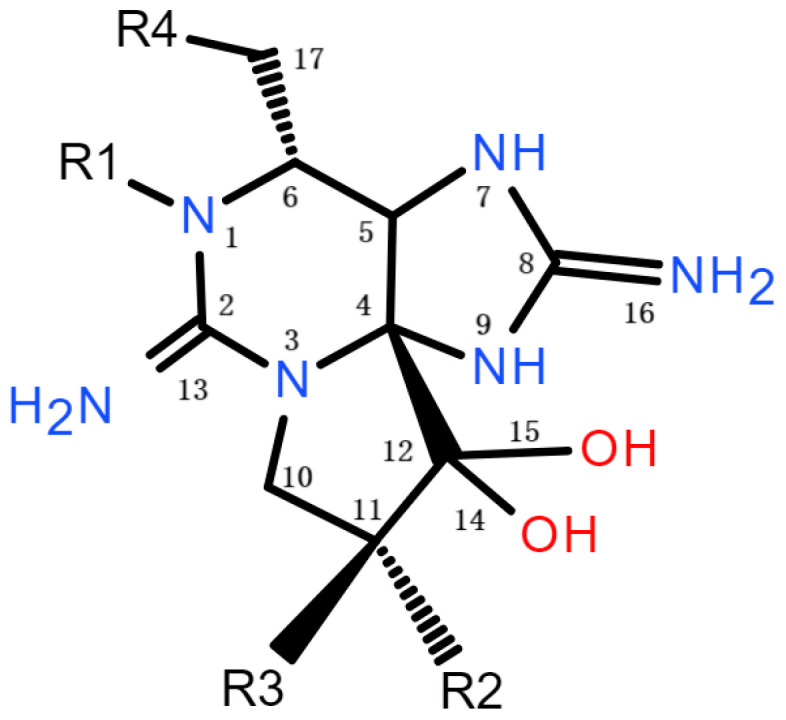
General chemical structure of PSP toxins.

PSP toxins are a group of neurotoxic alkaloids, more than 60 of which have been identified to date and are widely distributed in marine environments [78,79]. These analogs are generally classified into three major groups based on their distinct chemical structures at the R4 site (see Figure 2): carbamate toxins (e.g., GTX1&4, GTX2&3, STX, neoSTX), N-sulfocarbamoyl toxins (e.g., C1&2, C3&4, GTX5, GTX6), and decarbamoyl toxins (e.g., dcGTX1&4, dcGTX2&3, dcSTX, dcneoSTX) [80]. The toxicity of PSP toxins is ranked from highest to lowest as follows: carbamate toxins, decarbamoyl toxins, and N-sulfocarbamoyl toxins [81]. Within these PSP toxins, STX is the most potent neurotoxin, with an LD_50_ value of 10 µg kg^−1^ in mice (intraperitoneally) [82]. The minimum lethal dose (MLD) of PSP toxins for humans is reported to be 3000 mouse units (MUs), which is primarily determined based on death cases caused by PSP toxins. A single MU of PSP toxins corresponds to the quantity of toxin capable of killing a 20 g male ddY strain mouse within 15 min following intraperitoneal injection [83]. According to the above data, the PSP toxin-related toxicity characterized by MUs is presented in Table 1. Currently, several marine dinoflagellates, such as *Pyrodinium bahamense*, *Gymnodinium catenatum*, and various species of *Alexandrium*, along with some freshwater cyanobacterial species, have been identified as producers of PSP toxins [84,85,86]. PSP toxins in those marine dinoflagellates exist almost exclusively as β-epimers (GTX3&4, C2&4), while α-epimers (GTX1&2, C1&3) predominate in the shellfish profile, with an epimer α:β ratio close to 3:1 at equilibrium [82]. In addition to common PSP toxin derivatives, many new C-11 hydroxyl metabolites (so-called M-toxins) of PSP toxins have been discovered in contaminated shellfish. Trace amounts of such substances have also been detected in some strains of microalgae that produce PSP toxins [87]. M-toxins are regarded as low-toxic derivatives of typical PSP toxins based on their structure–activity relationships [88]. Considering that M-toxins quickly accumulate in high concentrations within shellfish following the ingestion of PSP toxin-producing microalgae, the M-toxins are regarded as metabolic products of PSP toxins during shellfish detoxification [89]. The toxins M1&M3 and M7&M9 can be transformed from C1&2 and C3&4, respectively, while M2&M4&M6 and M8&M10 can be transformed from GTX2&3 and GTX1&4, respectively. The transformation rates of C&2 and GTX2&3 are significantly higher than those of C3&4 and GTX1&4, and the metabolic transformation of C1&2 to M1&3 occurs most rapidly, which is also faster than the production rate of other M-toxins in scallops and mussels [90]. Furthermore, a new class of PSP toxins, referred to as GC toxins, has been found in *G. catenatum*, which contain hydroxybenzoate R4 substituents [91]. Unlike the hydrophilic nature of the three main classes of PSP toxins, the hydroxybenzoate side chains in GC toxins introduce a degree of lipophilicity [92]; however, the exact toxicity of GC toxins has not yet been determined.

#### 2.2.2. Main Biological Vectors of PSP Toxins

##### Bivalves

Although PSP toxins transfer through the food web and accumulate from zooplankton to whales [93,94], this review only focuses on the vectors known to pose a potential public health risk to human consumers. The traditional route of PSP toxins through the food web involves filter-feeding mollusks, particularly bivalves, which ingest and concentrate PSP toxins and associated HAB species [79,95,96,97]. PSP toxins exert detrimental impacts on various physiological functions of bivalves, including reduced clearance rates, adductor paralysis, mantle retraction, decreased valve closure response, and even death [98,99,100,101]. In addition, PSP toxins show obvious immunotoxicity to bivalves, mainly manifested as infiltration of hemocytes (especially in the connective tissues surrounding the digestive gland), suppression of hemocyte phagocytic activity, and induction of apoptosis [100,101,102,103,104,105]. 

Despite these adverse biological effects, bivalves were initially observed to survive and thrive during the periods of PSP toxin-producing dinoflagellate blooms and exhibited an ability to accumulate and tolerate higher concentrations of PSP toxins compared to humans [106]. Similar to tetrodotoxin (TTX), PSP toxins attack the nervous system by blocking sodium channels on the nerve membrane and inhibiting action potential conduction [107]. Voltage-gated sodium channel 1 (Nav1) is the major sodium channel in the animal nervous system and the target of PSP toxins [106,108]. Li et al. (2017) analyzed the scallop genome and identified two sodium channel genes, Nav1 and Nav2. Further analysis revealed the presence of a potential antitoxin T mutation at 1425 of Nav1 in scallops [106], which, in rats, corresponds to a 15-fold increase in resistance to saxitoxin (STX) (the most toxic PSP toxin) and a similar 15-fold increase in resistance to TTX [107,109]. Furthermore, analysis of Nav1 in *C. gigas* and the Atlantic clam *Solemya velum* revealed the presence of a Q mutation at site 945, which has been demonstrated to confer a 19,880-fold increase in resistance to STX in rats [110]. These findings suggest that the two novel mutations on Nav1 can enhance toxin resistance in other organisms and that their presence in bivalves may account for the ability to tolerate PSP toxins. 

It is found that the hepatopancreas and kidneys are the organs with the highest PSP toxins concentrations in *C. farferri* after exposure to PSP toxin-producing microalga *A. minutum.* Interestingly, there is little expression of sodium channel genes in either tissue [106], implying that bivalves may possess alternative mechanisms to tolerate high concentrations of PSP toxins. *A. minutum* showed greater toxicity to the kidney than the hepatopancreas, possibly due to the conversion of GTX into highly toxic STX in the kidney [106]. The elevated expression of the cytoplasmic sulfotransferase (*Sult*) gene, which likely mediates the transfer of sulfate groups from donor molecules (such as GTX) to various receptor molecules, endogenous metabolites, and xenobiotics [111], corresponds with the transformation of PSP toxins in the kidney. Furthermore, the *Sult* gene family is notably expanded in the *C. farreri* genome, with 83 genes compared to 26 in oysters, 31 in pearl oysters, 13 in humans, and 8 in flies [106]. All these findings suggest that the expanded *Sult* genes may be responsible for converting GTX into the more potent STX, potentially endowing scallops with a robust deterrent mechanism against predation. In addition, the expression of detoxification- and antioxidant-related genes such as superoxide dismutase (*SOD*) [112], *GST* [113], ABC transporters [114], and heat shock proteins [115] was markedly induced in bivalves as a response to counter the adverse impacts of PSP toxins.

Unfortunately, the mass mortality of bivalves has been increasing globally in recent years. PSP toxins have been linked to the mass mortality of many bivalve species [101]. While there is no definitive evidence that HABs directly cause mortality events in bivalves [116,117,118], recent studies have found that anthropogenic global factors such as global warming, ocean acidification, and widespread microplastic pollution may exacerbate bivalves’ susceptibility to toxigenic HABs (including PSP toxins and DSP toxins) [119,120,121,122]. These factors could potentially explain the mass mortality of bivalves caused by HABs in recent years.

##### Fish

Recently, the rising global demand for protein has spurred the expansion of aquaculture worldwide, with fish being one of the most widely farmed species in this sector [123]. Given the impact of HABs on human health, the action of shellfish has been thoroughly documented, prompting the establishment of marine toxin monitoring programs in most coastal nations [3]. However, since fish are not the primary focus of these monitoring efforts, data on the bioaccumulation of toxins in fish is comparatively scarce. PSP toxins can accumulate in fish that prey on zooplankton and subsequently exert detrimental effects on these fish [96,124]. Early field observations indicate that poisoning symptoms in fish affected by PSP toxins include loss of balance, lateral or inverted swimming, and swimming with open mouths [125,126]. Upon exposure to extracts from PSP toxin-producing algae via gavage, fish engaged in rapid circular swimming in confined spaces for a brief duration before ultimately becoming motionless on the bottom, with some individuals displaying significantly reduced respiratory rates [127]. Salierno et al. (2006) found that killifish (*Fundulus heteroclitus*) subjected to high doses of STX showed similar toxic symptoms, possibly due to the inhibited expression of c-FOS protein in the anterior and posterior lobes, which govern swimming and other motor functions [128]. In addition, PSP toxins have immunotoxic and hepatotoxic effects on fish and can induce oxidative stress that damages the gills, thereby increasing the oxygen consumption and demand of fish [129,130,131]. The similarity in poisoning symptoms observed among different wild fish species and those in laboratory conditions suggests that the physiological response to PSP toxins may be a common occurrence across the marine fish population. Moreover, Bianchi et al. (2021) found that other undefined soluble extracellular compounds produced by *Alexandrium* spp. were highly ichthyotoxic to fish [132]. This suggests that future studies of PSP-associated species should broaden their scope to include these extracellular substances.

#### 2.2.3. Mechanism of PSP Toxin Toxicity to Humans

PSP toxins pose a serious threat to public health primarily through contamination of the human food supply chain [133]. Symptoms of PSP begin with numbness and tingling in the lips and mouth and may progress to muscle weakness, ataxia, neurological symptoms, and, in severe cases, muscle paralysis and possibly death from respiratory paralysis [70,134,135]. Voltage-gated sodium channels serve as the molecular targets for PSP toxins, which share a common structural motif consisting of a primary α subunit with a molecular size of 220–260 kDa and one or two auxiliary β subunits with a molecular size of 33–36 kDa [136,137,138]. PSP toxins act by reversibly binding to sodium ion channels in the neural membrane, thus interrupting neuronal transmission and inducing systemic neurological dysfunction [135,139,140]. As mentioned above, unlike the susceptibility in humans, natural selection pressure has led to mutations in sodium channels within bivalves [106,107], resulting in tolerance to PSP toxins.

### 2.3. ASP Toxins

#### 2.3.1. ASP Toxins: Physicochemical Properties and Structure

**Figure 3:**
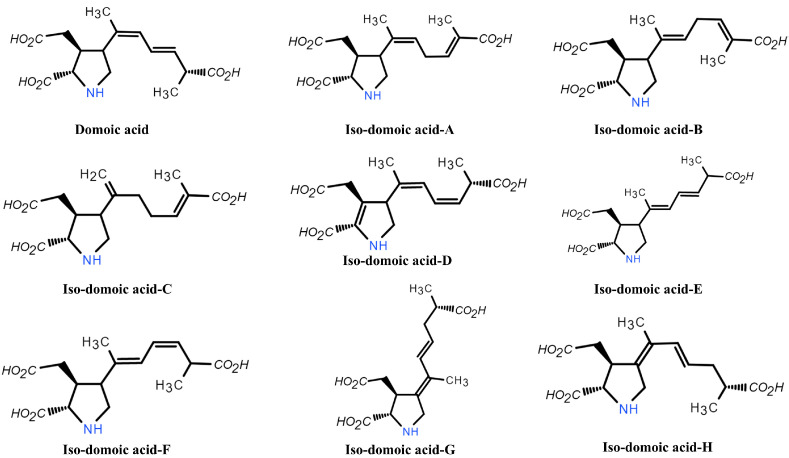
General chemical structure for DA and its isomers.

ASP toxins include domoic acid (DA) and its isomers, such as isodomoic acid. DA is a naturally occurring crystalline, water-soluble, acidic amino acid with a secondary amino group, and the LD_50_ of DA in mice (intraperitoneally) was assessed as 2.4 mg kg^−1^ [17,141]. ASP toxins are synthesized exclusively by specific species of diatoms, mainly the genus *Pseudo-nitzschia*, as well as certain members of the *Nitzschia* and *Amphora* genera, red alga *Chondria armata*, and other related species [17,142]. DA consists of a proline ring, three carboxyl groups, and one amino group. Its high polarity is due to the presence of three carboxyl groups and one amino group, with p*K*_a_ values of 1.85, 4.47, 4.75, and 10.60, respectively, at 20 °C. Therefore, depending on pH, DA may exist in five different states of charge, ranging from −3 to +1, which can affect its retention in different chromatographic systems [143]. The side chain of the DA molecule encompasses two conjugated double bonds, giving DA the ability to absorb ultraviolet light. At neutral pH, the maximum emission wavelength is 242 nm, which can be utilized for the detection of DA through liquid chromatography [144]. These structural features of DA are directly associated with the interaction with glutamate receptors and the toxicity of the molecule [145]. Despite its relative stability at room temperature [143], DA can undergo substantial decomposition under high temperatures (>50 °C) and in highly acidic (pH ≤ 2) or alkaline (pH ≥ 12) conditions [144]. Within shellfish tissue, however, the situation is slightly different. McCarron and Hess (2006) indicated that conventional cooking and pressure sterilization at 121 °C in mussel tissue only decreases the total DA concentration by approximately 3%, suggesting that cooking does not significantly improve the safety of DA-contaminated shellfish [146]. Moreover, the chemistry properties of DA and its analogs and geometrical isomers of iso-domoic acid A, B, and C were investigated in *C. armata* [147]. Three other iso-domoic acids, D, E, and F were isolated from plankton cells and shellfish tissues [148]. In addition, two new isomers of DA from *C. armata*, namely, iso-domoic acid G and H, were isolated in 1997 (see Figure 3) [147]. Iso-domoic acids are secondary components relative to DA and are not always found in bivalves contaminated with ASP toxins [149]. When exposed to ultraviolet light or heat, DA can be converted to iso-domoic acid [150,151]. Therefore, it has been proposed that domoic acid may be a biosynthetic product that is subsequently converted to iso-domoic acid under suitable environmental conditions [150].

#### 2.3.2. Main Biological Vectors of ASP Toxins

##### Bivalves

Bivalves are the main carriers of DA, and most DA in these organisms is not metabolized and remains unchanged when excreted [152]. The accumulation kinetics and depuration rates of DA vary significantly among different shellfish species [153,154]. Based on this, bivalves can be categorized into two groups: rapid and slow detoxifiers of DA. Rapid detoxifiers can eliminate toxins within days to weeks, including some mussel species, such as the mussels *M. galloprovincialis* [155], *M. edulis* [156], *M. californianus* [157], and *P. canalicus* [158], clams, such as *Mesodesma donacium* [159] and *Ruditapes philippinarum* [160], oysters, such as *C. virginica* [161] and *C. gigas* [162], and pectinids, like *Argopecten purpuratus* [153]. The slow detoxifiers, which may take months or even years to depurate DA, mainly include some commercially important bivalves such as *Pecten maximus* [163], *Placopecten magellanicus* [164], *Siliqua patula* [165], and *Spondylus cruentus* [166]. However, the mechanisms underlying the extensive variability in DA accumulation and detoxification kinetics in bivalves remain unclear. 

Trainer and Bill (2004) suggested that the expression of low-affinity glutamate receptors across all tissues and the selective expression of high DA capacity sites in tissues such as the siphon might be responsible for the tissue-specific retention of ASP toxins in species like *S. patula* [167]. Mauriz and Blanco (2010) proposed that the lack of effective membrane transport proteins for toxin excretion could account for the high accumulation and/or slow detoxification rate of DA in *P. maximus* [168]. In addition, by using a novel immunohistochemical technique based on an anti-DA antibody, García-Corona et al. (2022; 2024a) observed that in the digestive glands of species such as *P. maximus*, *Aequipecten opercularis*, and *Crepidula fornicate*, most DA staining was confined to small autophagic vesicles within the cytoplasm of digestive cells during early autophagy and in the post-digestive residues remaining in the distal cytoplasmic region of digestive cells during late autophagy [169,170]. García-Corona et al. (2024b) further analyzed the dynamics of DA subcellular localization in the digestive glands of *P. maximus*, suggesting that autophagy may be a contributing factor to the long-term retention of DA in *P. maximus* [171].

Several studies have shown that exposure to ASP toxin-producing microalgae or DA can trigger a range of detrimental physiological alterations in bivalves. For instance, *M. edulis* exhibited transient DNA damage after DA injection [172]. *M. californianus* developed mild respiratory alkalosis upon exposure to ASP toxin-producing microalgae *P. nitzschia* [173]. Similarly, *C. gigas* displayed transient respiratory acidosis, hypoxia, and an increase in hemocyte activity and count following exposure to *P. nitzschia* [162]. Dissolved DA can negatively affect the growth and survival of *P. maximus* larvae and impair immune function and induce oxidative stress in *A. irradians* [174,175,176]. Furthermore, transcriptomic analysis of the digestive glands in *M. galloprovincialis* and *A. opercularis* exposed to *P. nitzschia* revealed differential expression of genes involved in antioxidant, metabolic detoxification, transmembrane transport, and immune processes [177,178]. Oxidative stress is the main effect observed in other invertebrates exposed to DA [179,180]. Song et al. (2020) reported that exposure to pure DA induces oxidative stress in bay scallops, disrupts their metabolic processes, and negatively affects their defense response [181]. These findings suggest that oxidative stress may contribute significantly to the toxicity of DA in bivalves. Despite being a neurotoxic amino acid, available data suggest that DA has no significant neurotoxic effect on bivalves [172].

##### Fish

Although planktivorous fish can accumulate considerable amounts of DA during toxic *P. nitzschia* blooms [182], there is no evidence that fish are affected under natural exposure conditions. Fish exhibit neurotoxic symptoms when DA is administered intraperitoneally, whereas no such effects are observed when the toxins are orally ingested, which is the most ecologically relevant exposure route [183]. Even though oral DA builds up in the brain, liver, and kidneys of species such as salmon (*Oncorhynchus kisutch*) [184], rainbow trout (*Oncorhynchus mykiss*) [185], and anchovy (*Engraulis mordax*) [186], they do not exhibit excitotoxic behaviors. Consequently, the prevailing dogma is that fish are relatively insensitive to DA acquired through ecological exposure routes [183,187], but the reasons for this are unclear.

#### 2.3.3. Mechanism of ASP Toxin Toxicity to Humans

The first case of human DA poisoning occurred in Canada in 1987, with clinical symptoms of acute poisoning such as confusion, disorientation, seizures, permanent short-term memory loss, and, in the most severe cases, even death, so it is known as ASP [188]. The structure of DA is similar to neurotoxin kainic acid (KA) and the endogenous neurotransmitters glutamate (Glu) and aspartate (Asp) [183,189]. Consequently, it is not surprising that DA interacts with glutamate receptors (GluR) in nerve terminals. Glu is the main excitatory neurotransmitter in the brain; however, excessive Glu can lead to neurodegeneration [190], seizures [191], and apoptosis [192,193]. For example, overactivation of endogenous Glu or Asp on GluR triggers a cascade of biochemical events that can lead to neuronal damage or cell death [194,195]. Additionally, the rigid structure of DA significantly reduces its mobility at glutamate receptor binding sites, thereby improving the binding efficiency [196]. DA-induced excitotoxicity acts in a manner similar to Glu and KA, with a cohesive effect on both sides of the synapse, resulting in depolarization and the release of Glu into the synaptic gap [197]. The stimulation by DA results in a rapid influx of Ca^2+^, transporting Glu-containing vesicles to the membrane surface and releasing them into the synaptic space via exocytosis. Excessive Ca^2+^ influx disrupts Ca^2+^-dependent cascades [189], ultimately leading to neuronal death, including membrane, cytoplasmic, and nuclear events that lead to neurotoxicity.

### 2.4. NSP Toxins

#### 2.4.1. NSP Toxins: Physicochemical Properties and Structure

**Figure 4:**
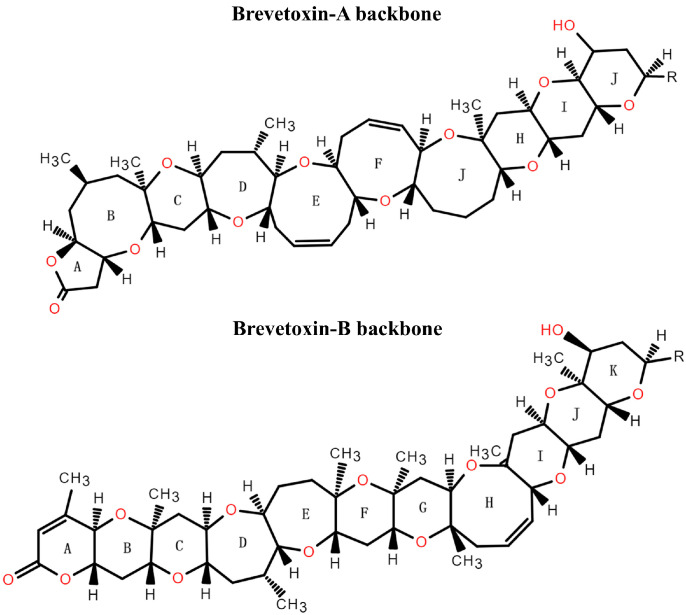
BTXs are based on two different structural backbones, which are perceived to be the two parent molecules, PbTx-1 (brevetoxin-A backbone) and PbTx-2 (brevetoxin-B backbone).

NSP toxins are mainly composed of brevetoxins (BTXs), which are produced mainly by the marine dinoflagellate *Karenia brevis* [198,199]. In addition, some strains of *K. papilionacea* are very low producers of BTXs [200]. BTXs are a class of lipid-soluble cyclic polyether compounds, which can be divided into A-type and B-type based on their backbone structure [201,202]. A-type BTXs have a backbone structure consisting of ten trans-fused ether rings, while that of B-type BTXs is composed of eleven ether rings (see Figure 4) [203]. Through molecular modeling, Rein et al. (1994a, 1994b), as well as Gawley et al. (1995), found that the structures of PbTx-1 and PbTx-2 are relatively linear, with a bend approximately in the middle of the molecule, a lactone function in the A ring, a strict rigid region in the four terminal rings, a side chain at the molecular end that allows moderate modification, and a spacer region that separates the rigid region from the A ring lactone [204,205,206]. All natural BTXs and derivatives with full activity have all these characteristics [202]. BTXs can also be distinguished by side chains located in the tail region. Both A-type brevetoxin-1 (PbTx-1) and B-type brevetoxin-2 (PbTx-2) possess the same side chain, with a highly active α, β-unsaturated aldehyde group at the end of the side chain [203]. Of the BTXs group, PbTx-1 is considered to be the most potent, while PbTx-2 is the most abundant toxin produced by *K. brevis* observed to date [207].

#### 2.4.2. Main Biological Vectors of NSP Toxins

##### Bivalves

Several studies have shown that various bivalve species can accumulate PbTxs [208,209,210]. In general, bivalves do not appear to suffer adverse effects from the toxins [199,201], although some studies suggest that exposure to NSP toxins may affect the development and clearance of bivalve larvae [210,211]. Despite the limited studies on the molecular mechanisms underlying toxic responses in bivalves to NSP toxins, the metabolism of NSP toxins across bivalve species may partially explain the toxicological responses of NSP toxins in bivalves. Ishida et al. (1995) identified a conjugate of oxidized PbTx-2 with C-42 N-taurine in cockle *Austrovenus stutchburyi*, naming it PbTx-B1 [212]. Morohashi et al. (1995) determined that PbTx-B3 is formed from PbTx-2 through fatty acid esterification at the open D-ring and oxidation at the aldehyde terminus [213]. Subsequently, Murata et al. (1998) discovered a conjugate with S-cysteine (sulfoxide) at the C-50 S-cysteine in *P. canaliculus*, termed PbTx-B2 [214]. Morohashi et al. (1999) further identified conjugates of the PbTx-B2 cysteine sulfoxide moiety with *N*-myristoyl and *N*-palmitoyl groups in *P. canaliculus* and named it PbTx -B4 [215]. Furthermore, the main metabolites of PbTx-2 identified in Easter oysters include the reduction derivative PbTx-3, the cysteine sulfoxide conjugate PbTx-B2, and the newly identified S-deoxy-PbTx-B2, which is easily oxidized to PbTx-B2 [216]. Ishida et al. (2004) characterized the oxidized derivative of PbTx-2, specifically, the terminal aldehyde in cockle, and named it PbTx-B5 [217]. The discovery of a large number of PbTx metabolites in shellfish increases the difficulty of detecting these toxins. However, the current studies suggest that PbTxs ingested by humans through shellfish are metabolites of PbTxs, rather than the parent NSP toxins from algae [202,218], which undoubtedly emphasizes the importance of detecting these metabolites.

The depuration time of PbTxs in bivalves varies but is usually within 2 to 8 weeks, although there are records of PbTxs being retained for much longer (nearly a year) [216,219,220]. Given that PbTxs can be metabolized in various molluscan shellfish, as mentioned above, the metabolism of PbTxs in shellfish may be partially responsible for their low impact on shellfish. The limited acute animal toxicity data of PbTxs and their metabolites is summarized in Table 2. The 24 h LD_50_ of PbTx-2 administered intraperitoneally in mice was 200 µg kg^−1^ body weight, whereas the oral toxicity of PbTx-2 was significantly lower in comparison. With a few exceptions, the metabolism of PbTx-2 mitigates its acute toxicity under the same experimental conditions. Specifically, the 24 h LD_50_ of PbTx-3, S-deoxy-PbTx-B2, and PbTx-B2 administered intraperitoneal (i.p.) in mice was 875, 211, and 400 µg kg^−1^ body weight, respectively. However, the current understanding of the mechanisms associated with the toxic response to PbTxs in bivalves remains limited.

##### Fish

Additionally, PbTxs are ichthyotoxins that typically kill millions of fish and affect hundreds of species [78,225,226]. Fish mortality typically results from the rupture of algal cells and the subsequent release of PbTxs, which are absorbed directly through the gill membranes [207]. However, some studies indicate that fish can endure HABs and act as a significant vector for the transfer of PbTxs to higher trophic levels [227,228]. Naar et al. (2007) found that PbTxs can accumulate in live fish via dietary exposure, and fish fed with PbTx-contaminated shellfish or *K. brevis* cultures remain healthy while accumulating high levels of PbTxs in their tissues [228]. Moreover, Griffin et al. (2023) demonstrated that Atlantic tarpon (*Megalops atlanticus*) is tolerant to *K. brevis* blooms [229]. Although, it has been suggested that species-specific susceptibility to both lethal and sublethal *K. brevis* effects is largely dependent on the intersection of innate tolerance, life history stage, dispersal ability, and behavioral ecology/environmental conditions, as well as bloom toxicity dynamics and distribution [230], and even the synergistic interaction between ROS (superoxide anion) and polyunsaturated fatty acids (PUFAs) [231]. To date, these contradictory findings have not been adequately explained. Therefore, this emphasizes the need to better assess the role of fish in the transfer of PbTxs, including their ability to metabolize PbTxs. However, compared to the advances in PbTx metabolism in bivalves, the metabolic fate of PbTxs in fish remains largely unexplored.

#### 2.4.3. Mechanism of NSP Toxin Toxicity to Humans

NSP usually occurs after consumption of bivalve mollusks contaminated with BTXs [20]. The characteristic clinical features of NSP mainly involve the gastrointestinal tract and the nervous systems, and the nervous system manifestations last longer than the gastrointestinal disturbances [232,233]. Specifically, these symptoms include nausea, vomiting, abdominal pain, and diarrhea, as well as partial limb paralysis, slurred speech, dizziness, ataxia, and a generalized loss of coordination [199,234]. The neurotoxic effects of BTXs have been widely substantiated, mainly due to their high affinity with the voltage-gated sodium channel (VGSC) receptor site 5 in nerve, skeletal, and cardiac cells [37], resulting in prolonged channel opening, particularly the inability of open channels to be inactivated, thereby leading to sustained depolarization of neurons [235,236]. This depolarization, in turn, triggers a cascade of downstream neurochemical effects, such as enhanced release of glutamate, overstimulation of N-methyl-d-aspartic acid (NMDA) receptors, and unregulated influx of Ca^2+^ into postsynaptic cells [237]. Ultimately, these events lead to the obstruction of ion conduction and action potential generation, resulting in the loss of neuromuscular function and muscle paralysis [238]. Given the similarity between the targets of BTXs and PSP toxins, the insensitivity of bivalves to BTXs may also be attributed to mutations in sodium channel sites in these organisms. However, this hypothesis requires further investigation to be substantiated. 

### 2.5. CP Toxins

#### 2.5.1. CP Toxins: Physicochemical Properties and Structure

**Figure 5:**
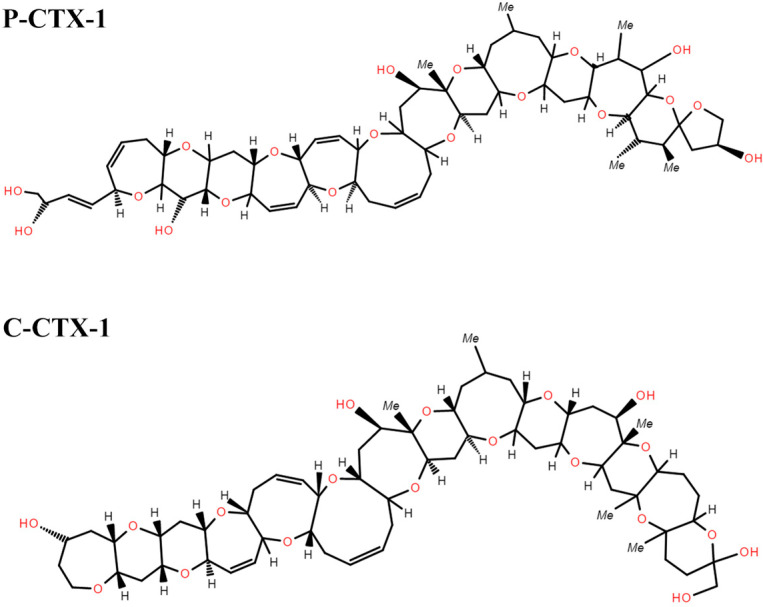
General chemical structure of Pacific ciguatoxin-1 (P-CTX-1) and Caribbean ciguatoxin-1 (C-CTX-1). The major CTXs from the Indian region (I-CTXs) have a similar structure to C-CTX-1.

CP toxins, including ciguatoxins (CTXs), maitotoxins (MTXs), gambierol, and related compounds, are associated with foodborne ciguatera fish poisoning [239]. These toxins are produced by the dinoflagellates *Gambierdiscus* and *Fukuyoa* (more than 18 species produce MTX, but only 3 or 4 produce CTX) [240]. They are bioactive molecules consisting of ether rings, with significant differences in carbon number, structure, and mechanism of action. Among them, CTXs are polyether neurotoxins that can directly cause CP and are currently one of the most studied CP toxins [239]. They consist of a ladder-like structure composed of 13 or 14 ether rings and can be classified into Pacific (P-), Caribbean (C-), and Indian Ocean (I-) groups based on the number of rings, backbone structure, and region (see Figure 5) [241]. Although the geographic classification of CTXs is still in use, it does not adequately characterize the diversity of CTXs, and classification and nomenclature based on structural features have evolved. Structurally, CTXs can be categorized into three types of molecules: the oxopene (CTX1B type), oxocene (CTX3C type), and Caribbean/Indian CTXs structures [239,242,243]. The first two are commonly associated with fish and microalgae in the Pacific, and the main CTX toxins include P-CTX-1, P-CTX-2, and P-CTX-3. The derivative found in Pacific fish, CTX1B, is one of the most toxic analogs; hence, the Toxicity Equivalence Factor (TEF) recommended by the European Food Safety Authority (EFSA) is assigned with CTX1B as the reference (TEF = 1). CTX-2 and CTX-3 both have a TEF of 0.3, while CTX3C has a TEF of 0.2, and C-CTX-1 has a TEF of 0.1. These values were determined by acute toxicity testing via i.p. in mice [60,244]. However, a recent study by Raposo-García et al. (2023) showed that the effects of the analogs CTX3C and C-CTX1 were only 0.3 times lower than that of the Pacific analog CTX1B, both in terms of cytotoxicity and reduced sodium current intensity [245]. These data contradict the statements of the EFSA and the U.S. Food and Drug Administration (FDA) that the relative potency of C-CTX1 is 10 times lower than that of the Pacific analogs. Therefore, it is necessary to re-evaluate the relative toxicity and potency of ciguatoxins to determine the safety limits for these emerging toxins in commercialized fish.

#### 2.5.2. Main Biological Vectors of CP Toxins

CTXs originate from the benthic dinoflagellate genus *Gambierdiscus*, which is mainly found in tropical and subtropical seas. When toxin-producing algae are consumed by herbivorous fish, which are consumed by carnivorous fish, CTXs are transferred through the food web, ultimately resulting in poisoning incidents when humans consume fish contaminated with CTXs [244,245,246,247,248]. A survey of CTXs in a coral reef system showed that P-CTX was detected in 54% of herbivorous fish (total P-CTXs < 0.500–1670 pg·g^−1^ wet weight (ww)), in 72% of omnivorous fish (<0.500–1810 pg·g^−1^ ww), and in 76% of predatory fish (<0.500–69,500 pg·g^−1^ ww) [249].

Numerous studies have confirmed that P-CTX-1 can induce developmental, reproductive, and cardiovascular toxicity in fish [250,251,252]. Furthermore, juvenile fish exposed to P-CTX-1 exhibit reduced, delayed, or paralyzed responses to external stimuli, indicating that P-CTX-1 may impair the physiological behaviors of fish, such as foraging and evading predators [251]. Yan et al. (2017) found that injecting P-CTX-1 into *Oryzias melastigma* embryos resulted in malformations, visceral damage, immune dysfunction, and muscle physiological changes. Subsequent 21-day exposure reduced spawning and hatching rates in the progeny [253]. Jiang et al. (2012) conducted proteomic analysis on fish in CTX-contaminated fields and found that CTXs disrupted the influx/efflux of Na^+^ and Ca^2+^ in fish livers and interfered with various biological functions, such as signal transduction, metabolic processes, detoxification, anti-apoptosis, and immune defense [250]. Li et al. (2022) showed that direct or indirect exposure to CTX-producing microalga *G. caribaeus* affected the swimming behavior of *O. melastigma*, characterized by reduced swimming ability and abnormal swimming patterns. Further transcriptomic analysis revealed that *G. caribaeus* ingestion could significantly affect energy metabolism, induce immune stress, and up-regulate the expression of pro-inflammatory factors, causing liver tissue damage [254]. These findings indicate a range of adverse effects of CTXs on fish. However, Xu et al. (2021) found that *G. caribaeus* from Weizhou Island, China, has relatively low toxicity, yet fish contaminated with CTXs exceeded regulatory thresholds, suggesting that the long-term presence of low-toxicity *G. caribaeus* may lead to the accumulation of CTXs in fish [255]. The ability of fish to accumulate CTXs implies that they may have some resistance. Consistent with the findings of Xu et al., juvenile *Naso brevirostris* exposed to a gel food embedded with CTX-producing microalga *G. polynesiensis* for four months exhibited no signs of intoxication [256], which seems to contradict the toxic effects of CTX on fish mentioned above [250,251,252,253,254]. In addition to species-specific tolerance to CTXs, the discrepancy in outcomes may be related to the microalgae species/strains used in the experimental test. It has been demonstrated that the toxicity of specific *Gambierdiscus* strains is 100 times higher than that of other strains within the same species [257,258]. This variability seems to partially account for the conflicting results. 

Currently, CP is an emerging threat in Europe. The first outbreak was recorded in 2004 in the Canary Islands of Spain [259]. Since then, Portugal reported its first case in 2008, when 11 of 16 crew members on a fishing vessel developed CP symptoms after consuming 30 kg amberjack (*Seriola* sp.) caught in the Selvagens Islands [260]. In the same year, CP symptoms were also observed in 20–30 individuals who consumed a smaller amberjack (*Seriola rivoliana*) that was purchased in the Canary Islands but caught in the Selvagens Islands [261]. Recently, red porgy (*Pagrus pagrus*) from the Selvagens Islands was found to have toxicity levels as high as 0.76 µg C-CTX1 kg^−1^ [262]. In addition, other mid-trophic level marine fish species, including zebra bream (*Diplodus cervinus*) and barred hogfish (*Bodianus scrofa*), also showed CTX-like toxicity up to 0.75 µg C-CTX1 kg^−1^. Furthermore, several fish species have the potential to act as vectors for the transmission of ciguatoxin to humans, including the parrotfish (*Sparisoma cretense*), the grey triggerfish (*Balistes capriscus*), and the blacktail comber (*Serranus atricauda*), highlighting that the risk of CP is not limited to top predators [263]. More notably, CTX-associated mass spectrum signals and ion fragmentation patterns were detected in random samples of benthic organisms, including the starfish *Ophidiaster ophidianus* off the southeast coast of Madeira Island [264]. Moreover, invertebrates, such as shellfish, could potentially be responsible for CP [265]. Ciguatera shellfish poisoning events involving giant clams (*Tridacna maxima*) are reported occasionally in Pacific island communities, and even after purification, the CP toxin in *T. maxima* will not be eliminated [266]. These findings highlight the necessity for further investigation of the toxic response and transfer kinetics of CTX in benthic organisms.

#### 2.5.3. Mechanism of CP Toxin Toxicity to Humans

CP is well-known in tropical regions, but with global warming, the risk of CP is increasing with the geographical spread of CP toxin-producing algae [267,268]. CP can cause neurological, gastrointestinal, and cardiovascular symptoms, depending on the amount and type of toxin ingested [247,269,270], and in severe cases, CP can be fatal [271]. P-CTX-1 as low as 0.08–0.1 µg kg^−1^ in fish meat can pose a risk to human health [248]. CP symptoms usually appear within 12 h of consuming toxic fish and begin with severe gastrointestinal issues (nausea, vomiting, diarrhea, abdominal pain) and typically subside within 24 h [272]. Cardiovascular issues (usually a combination of bradycardia and hypotension) and/or neurological symptoms (generally confusion, memory impairment, difficulty concentrating, depression or irritability, and anxiety) may also accompany this acute episode [246,273,274,275]. 

CTXs activate VGSC in cell membranes, thereby increasing sodium permeability and depolarizing nerve cells. This nerve cell depolarization is thought to cause a range of neurological symptoms associated with CP [276]. Despite detailed case reports, there is a lack of accurate identification and comprehensive understanding of the characteristics of CP, and no definitive biomarkers have been developed to diagnose CTX exposure in humans. Currently, the “gold standard” for CP diagnosis includes the detection of CTXs in relevant fish species through appropriate testing methods, such as animal mortality tests, biological methods (cytotoxicity assays, receptor binding assays, and immunoassays), and chemical methods (HPLC with fluorescence detection, LC-MS/MS) [277], along with symptoms consistent with CP.

## 3. Conclusions

Collectively, shellfish and fish are the main vectors of HAB toxins. Unlike the susceptibility of humans to HAB toxins, shellfish and fish may have developed some tolerance due to natural selection pressures, though the underlying mechanisms of this tolerance remain poorly understood. Since there are currently no antidotes for HAB toxins, symptom management and supportive care are the only treatment options available once a case is confirmed. In view of this, public health surveillance and intervention for HABs are critical to managing risks related to human health. Further studies are necessary to elucidate the underlying molecular mechanisms of fish and shellfish tolerance to HABs and related toxins, identify key biomarkers for HAB occurrence, develop rapid monitoring methods for toxins, especially real-time online monitoring methods, and develop effective antidotes or scavengers to mitigate public health concerns arising from HABs. Effective public health interventions include but are not limited to enhancing environmental monitoring for HAB toxins, identifying biomarkers for human diseases, further describing HAB-associated syndrome characteristics, revising disease reporting and monitoring schemes, and promoting policy development. The current approach for monitoring HAB toxin levels in contaminated aquatic organisms is the most direct, mainly relying on LC-MS/MS, but the high time and financial costs associated, as well as the limited availability of the necessary equipment, pose a challenge to detection. The future direction is to initially assess the contamination of HAB toxins by rapidly detecting changes in molecular markers or target molecules sensitive to specific HAB toxins in fish or shellfish, followed by a detailed analysis of the accumulation of HAB toxins using LC-MS/MS to formulate targeted public health intervention policies. Additionally, there are currently no effective antidotes or toxin removal agents, but the results from some in vivo studies in aquatic organisms suggest that adding natural active substances such as curcumin and cinnamaldehyde can significantly reduce the accumulation of DSP toxins in mussels and alleviate damage by affecting CYP3A4 in mussels, which has been proven to be involved in the metabolism of DSP toxins in shellfish [278,279]. Therefore, identifying the key biomarkers for the occurrence of HABs can not only effectively reduce algal toxins in aquatic organisms but also reduce the occurrence of human poisoning.

## Figures and Tables

**Table 1:** PSP toxin components and their specific toxicities (MU/μmol) adapted from [82].

PSP Toxins		R1	R2	R3	R4	Toxicity
Carbamate toxins	STX	H	H	H	CONH_2_	2483
	neoSTX	OH	H	H	CONH_2_	2295
	GTX1	OH	OSO_3_^−^	H	CONH_2_	2468
	GXT2	H	OSO_3_^−^	H	CONH_2_	892
	GXT3	H	H	OSO_3_^−^	CONH_2_	1584
	GXT4	OH	H	OSO_3_^−^	CONH_2_	1803
N-sulfocarbamoyl toxins	GTX5	H	H	H	CONHSO_3_^−^	160
	GXT6	OH	H	H	CONHSO_3_^−^	180
	C3	OH	OSO_3_^−^	H	CONHSO_3_^−^	33
	C1	H	OSO_3_^−^	H	CONHSO_3_^−^	15
	C2	H	H	OSO_3_^−^	CONHSO_3_^−^	239
	C4	OH	H	OSO_3_^−^	CONHSO_3_^−^	143
Decarbamoyl toxins	dcSTX	H	H	H	H	1274
	dcneoSTX	OH	H	H	H	33
	dcGTX2	H	OSO_3_^−^	H	H	1617
	dcGTX3	H	H	OSO_3_^−^	H	1872

**Table 2:** Acute toxicity of BTXs in mice.

BTXs	Route	Toxicity, μg kg^−1^ Body wt.	Reference
PbTx-2	LD_50_, 24 h, i.p.	200	[221]
	LD_50_, 24 h, oral	6600	[222]
PbTx-3	LD_50_, 24 h, i.p.	875	[222]
	LD_50_, 24 h, oral	520	[221]
S-deoxy-PbTx-B2	LD_50_, 24 h, i.p.	211	[223]
PbTx-B1	MLD ^a^, <2 h, i.p.	50	[212]
PbTx-B2	LD_50_, 24 h, i.p.	400	[223]
	MLD, <1 h, i.p.	306	[214]
PbTx-B3	MLD, 24 h, i.p.	>300 ^b^	[213]
PbTx-B4	MLD, 6–24 h, i.p.	100	[215]
PbTx-B5	MLD ^c^, i.p.	300–500	[224]

a. Minimum lethal dose. b. No deaths recorded at 300 µg kg^−1^. c. Time to death not reported.

## Data Availability

Not applicable.
